# A Novel Predictive Model for Acute Kidney Injury Following Surgery of the Aorta 

**DOI:** 10.31083/j.rcm2502054

**Published:** 2024-02-04

**Authors:** Mingjian Chen, Sheng Zhao, Pengfei Chen, Diming Zhao, Liqing Wang, Zhaoyang Chen

**Affiliations:** ^1^Department of Surgery, Fuwai Hospital, Chinese Academy of Medical Sciences & Peking Union Medical College, 100037 Beijing, China; ^2^Department of Cardiology, Fuwai Hospital, State Key Laboratory of Cardiovascular Disease, National Center for Cardiovascular Diseases, Chinese Academy of Medical Sciences & Peking Union Medical College, 100037 Beijing, China; ^3^Cardiology Department, Heart Center of Fujian Province, Union Hospital, Fujian Medical University, 350000 Fuzhou, Fujian, China

**Keywords:** acute kidney injury, macrophage polarization, cytokine, predictive model, aortic surgery

## Abstract

**Background:**

Acute kidney injury (AKI) frequently occurs after aortic surgery and has a significant impact on patient outcomes. Early detection or prediction of AKI is crucial for timely interventions. This study aims to develop and validate a novel model for predicting AKI following aortic surgery.

**Methods:**

We enrolled 156 patients who underwent on-pump aortic surgery in our hospital from February 2023 to April 2023. Postoperative levels of eight cytokines related to macrophage polarization analyzed using a multiplex cytokine assay. All-subset regression was used to select the optimal cytokines to predict AKI. A logistic regression model incorporating the selected cytokines was used for internal validation in combination with a bootstrapping technique. The model’s ability to discriminate between cases of AKI and non-AKI was assessed using receiver operating characteristic (ROC) curve analysis.

**Results:**

Of the 156 patients, 109 (69.87%) developed postoperative AKI. Interferon-gamma (IFN-γ) and interleukin-4 (IL-4) were identified as candidate AKI predictors. The cytokine-based model including IFN-γ and IL-4 demonstrated excellent discrimination (C-statistic: 0.90) and good calibration (Brier score: 0.11). A clinical nomogram was generated, and decision curve analysis revealed that the cytokine-based model outperformed the clinical factor-based model in terms of net benefit. Moreover, both IFN-γ and IL-4 emerged as independent risk factors for AKI. Patients in the second and third tertiles of IFN-γ and IL-4 concentrations had a significantly higher risk of severe AKI, a higher likelihood of requiring renal replacement therapy, or experiencing in-hospital death. These patients also had extended durations of mechanical ventilation and intensive care unit stays, compared with those in the first tertile (all *p* for group trend <0.001).

**Conclusions:**

We successfully established a novel and powerful predictive model for AKI, and demonstrating the significance of IFN-γ and IL-4 as valuable clinical markers. These cytokines not only predict the risk of AKI following aortic surgery but are also linked to adverse in-hospital outcomes. This model offers a promising avenue for the early identification of high-risk patients, potentially improving clinical decision-making and patient care.

## 1. Introduction

Acute kidney injury (AKI) is one of the most common complications following aortic surgery and elevates the risks for: postoperative mortality, renal replacement therapy (RRT), prolonged intensive care unit (ICU) and hospital stay, higher medical costs, and a continued risk of death 10 years after surgery [1, 2, 3]. Early identification of high-risk AKI patients is crucial due to the condition’s rapid progression and poor prognosis. While serum creatinine and urine output are standard diagnostic markers, their limitations, their predictive value may be limited due to their delayed rise, and the fact that urine output may not be a reliable indicator during the polyuric phase [4, 5]. Therefore, alternative biomarkers based on the pathophysiological features of AKI for early risk identification are needed. In addition to some well-studied urinary biomarkers such as neutrophil gelatinase-associated lipocalin (NGAL), insulin-like growth factor binding protein-7 (IGFBP-7), and tissue inhibitor of metalloproteinase-2 (TIMP-2) [6, 7, 8, 9, 10, 11], blood biomarkers involved in the immune-inflammatory phase of AKI have also been identified and warrant further research.

Previous research has demonstrated that the innate immune response is closely linked to the pathogenesis of renal ischemia-reperfusion injuries [12]. Among the innate immune cells involved in this process, macrophages have been found to play a complex role throughout the development of AKI via their polarization to either a “pro-inflammatory” or “anti-inflammatory” phenotype [13]. Several animal studies have confirmed that macrophage polarization is involved in the initiation and repair of AKI, and influences the outcome [14, 15, 16]. Macrophage polarization can be induced by various cytokines such as interferon-gamma (IFN-γ) and interleukin-4 (IL-4), and polarized macrophages also release various inflammatory cytokines which exert specific effects [17, 18]. Therefore, the evaluation of macrophage polarization-related cytokines may also be valuable in predicting AKI following surgery. Previous studies have shown that macrophage polarization-related cytokines such as monocyte chemotactic protein-1 (MCP-1), interleukin-10 (IL-10), interleukin-6 (IL-6), and interleukin-1 receptor antagonist (IL-1RA) are associated with AKI in patients undergoing aortic surgery [19, 20].

Despite their potential, these biomarkers that were individually selected to predict AKI, have demonstrated limited sensitivity and specificity in clinical practice. Moreover, these existing studies were limited to specific aortic disease groups, lacking independent validation in broader populations. Therefore, utilizing a more comprehensive set of macrophage polarization-related cytokines in a cohort of patients with multiple aortic diseases, and integrating multiple cytokines into a simple model may improve the prediction of AKI risk assessment.

In this study, we conducted a systematic analysis of eight macrophage polarization-related cytokines in plasma samples from adult patients who underwent aortic surgery with cardiopulmonary bypass (CPB). We used all-subset regression to identify promising cytokines for inclusion in a model aimed at predicting postoperative AKI. In addition, we assessed the ability of each cytokine of interest to determine the risk of AKI as well as in-hospital outcomes.

## 2. Materials and Methods

### 2.1 Study Population and Data Collection

This single-center, retrospective, observational study was performed at the Fuwai Hospital (National Center of Cardiovascular Diseases, Beijing, China). We enrolled 156 adult patients who underwent aortic surgery with CPB at the Fuwai Hospital between February 2023 and April 2023. Exclusion criteria included: (1) patients under 18 years of age or over 80 years of age; (2) the presence of comorbidities including urinary tract infection or obstruction, or chronic kidney disease; (3) a recent history of a kidney transplant or dialysis; (4) the use of medications with nephrotoxic effects two weeks before surgery; (5) severe rheumatic immune disease; (6) immunodeficiency syndromes. We obtained the clinical data from the medical records. Demographic and preoperative data included age, sex, body mass index (BMI), comorbidities, preoperative cardiovascular status, preoperative serum creatinine (last value before cardiac surgery), and the diagnosis of aortic disease. Operative details included type of surgery, CPB time, and arterial clamp times. Postoperative serum creatinine was obtained within 48 hours of surgery. This research was approved by the Institutional Ethics Committee of Fuwai Hospital (No. 2023-2005) and was conducted under the tenets of the Declaration of Helsinki. Written informed consent was obtained from all patients before enrollment.

### 2.2 Specimen Collection and Measurement of Blood Macrophage Polarization-Related Cytokines

Blood samples were taken between 12 and 24 hours following surgery in the morning (8:00 AM) of the first postoperative day to evaluate cytokines. Using a multiplex Bio-Plex Pro Human Cytokines Assay (Bio-Rad, Hercules, CA, USA), eight macrophage polarization-related cytokines (including IFN-γ, IL-4, IL-6, IL-10, IL-1RA, interleukin-1-beta [IL-1β], interleukin-12p40 [IL-12p40], and tumor necrosis factor-alpha [TNF-α]) were measured following the manufacturer’s recommendations. Serum creatinine was measured using the hospital clinical laboratory’s standard analyzer.

### 2.3 Diagnostic Criteria of AKI and Outcome Definition

AKI was defined in accordance with the Acute Kidney Injury Network (AKIN) guidelines: a greater than 50% rise in serum creatinine, or a more than 0.3 mg/dL (26.5 mol/L) increase in serum creatinine within 48 hours of aortic surgery, in comparison to baseline [21]. Baseline was defined as the minimum creatinine level 24 hours before aortic surgery. The stages of AKI depended on the variations in serum creatinine from the baseline creatinine and are shown in **Supplementary Table 1**. Composite outcomes included RRT and/or in-hospital death. Length of ICU stay, length of hospital stay, and time spent on mechanical ventilation were considered to be associated outcomes.

### 2.4 Statistical Analysis

Unpaired Student’s *t*-test or the Mann-Whitney U test were used to compare continuous variables, and were reported as medians with interquartile ranges. The chi-squared test or Fisher’s exact test was used to compare categorical variables that were reported as numbers and proportions. Analysis of variance or the Kruskal-Wallis test, if applicable, was used to assess differences between the three groups. Using univariate logistic regression analysis, the correlation between each cytokine and AKI was determined. The logistic regression model contained all variables that met the criteria for statistical significance in the univariate logistic regression analysis. The variables were screened using all-subsets regression, with the best model assessed by adjusted r-squared and Bayesian information criterion (BIC). The area under the receiver operating characteristic curve (AUC), which equates to the C-statistic, was calculated to assess the model’s discriminating power. The Brier score was used to evaluate the model’s calibration. The model was internally validated via bootstrapping. The net benefit and improvement of the model over the clinical factor-based model were compared using decision curve analysis (DCA), net reclassification improvement (NRI), and integrated discriminant improvement (IDI). In each analysis, statistical significance was defined as 2-tailed *p* < 0.05. The data were analyzed using SPSS version 25.0 (IBMCorp., Armonk, NY, USA) and R statistical packages (The R Foundation; http://www.r-project.org; version 4.2.0), and the graphs were generated using GraphPad Prism 9.0 (GraphPad Software, San Diego, CA, USA).

## 3. Results

### 3.1 Clinical Characteristics of the Study Population

In our cohort of 156 adult patients undergoing aortic surgery, 109 (69.87%) developed postoperative AKI. Among them, 63 patients (40.38%) experienced moderate AKI (AKIN stage 1) and 46 (29.49%) had severe AKI (AKIN stage 2–3). Renal replacement therapy (RRT) was required for 18 patients (11.53%), and all 7 in-hospital deaths all occurred in the AKI group; there were no deaths in the non-AKI group. Baseline characteristics, intraoperative details, and perioperative outcomes are outlined in Table 1. There were no significant differences in age, sex, BMI, smoking, diabetes mellitus, hyperlipidemia, preoperative left ventricular ejection fraction, preoperative serum creatinine, arterial occlusion time, and hospital days between the AKI and non-AKI groups. In contrast, hypertension, cardiac function (as classified by the New York Heart Association), diagnosis of aortic disease, type of surgery, and CPB duration were significantly different between the two groups. Patients in the AKI group had an increased incidence of prolonged ventilation time and prolonged ICU stay. The distribution of surgical procedures is shown in **Supplementary Table 2**. Amongst the procedures, 47 (30.13%) were simple aortic root procedures, 18 (11.54%) were simple aortic arch procedures, 9 (5.76%) were simple descending aortic procedures, and 82 (52.57%) were combined procedures involving at least two sites in the aortic root, ascending aorta, arch, and descending aorta.

**Table 1.** **Baseline characteristics, intraoperative details, and perioperative outcomes**.

			Non-AKI group (n = 47)	AKI group (n = 109)	*p* value
			Number (Proportion)	Number (Proportion)	
			Median (Q1–Q3)	Median (Q1–Q3)	
Demographic data					
	Age, years		57.00 (48.00–63.00)	56.00 (47.00–65.50)	0.49
	Male, n %		34 (72.34%)	84 (77.06%)	0.52
	BMI, ${\mathrm{kg/m}}^{2}$		24.91 (22.59–27.68)	25.95 (23.12–27.55)	0.58
Comorbidities					
	Smoking, n %		24 (51.06%)	54 (49.54%)	0.86
	Diabetes Mellitus, n %		5 (10.63%)	5 (4.58%)	0.28
	Hyperlipidemia, n %		25 (56.81%)	49 (44.95%)	0.18
	Hypertension, n %		20 (42.55%)	80 (73.39%)	<0.001
Preoperative cardiovascular status					
	Preoperative LVEF, n %		61.00 (56.00–63.00)	60.00 (58.50–64.50)	0.66
	NYHA III–IV, n %		3 (6.38%)	24 (22.01%)	0.018
Baseline renal function					
	Preoperative serum creatinine, µmol/L		80.80 (75.00–97.17)	88.00 (75.90–103.60)	0.28
Disease diagnosis					0.005
	Aortic aneurysm, n %		30 (63.82%)	43 (39.44%)	
	Aortic dissection: Stanford A, n %		11 (23.40%)	55 (50.45%)	
	Aortic dissection: Stanford B, n %		1 (2.12%)	5 (4.58%)	
	Other, n %		5 (10.63%)	6 (5.50%)	
	Marfan Syndrome, n %		1 (2.12%)	2 (1.83%)	1.00
Operative details					
	Surgery types				<0.001
		Surgery simply referring to the aortic root, n %	25 (53.19%)	22 (32.83%)	
		Surgery simply referring to the aortic arch, n %	4 (8.51%)	14 (12.57%)	
		Surgery simply referring to descending aorta, n %	5 (10.63%)	4 (6.28%)	
		Combined surgery, n %	13 (27.65%)	69 (57.29%)	
	Concomitant surgery				
		CABG, n %	6 (12.76%)	21 (19.26%)	0.32
		Mitral or tricuspid valve surgery, n %	1 (2.12%)	9 (8.25%)	0.28
	CPB time, min		121.00 (81.25–152.25)	149.50 (120.50–201.75)	0.001
	CPB time >120 min, n %		15 (31.91%)	60 (55.04%)	0.008
	Artery clamp time, min		93.00 (61.50–122.50)	99.00 (73.00–133.00)	0.29
Outcomes					
	48 h highest serum creatinine, µmol/L		86.54 (76.20–105.08)	173.81 (146.87–227.74)	<0.001
	Hospitalization, days		15.00 (11.00–21.00)	15.00 (12.00–25.00)	0.42
	ICU time, days		3.00 (1.50–4.00)	5.00 (2.00–8.00)	0.005
	Ventilation, hours		16.00 (13.00–21.00)	25.00 (16.00–64.50)	0.001
	RRT, n %		0 (0.0%)	18 (16.51%)	0.003
	In hospital death, n %		0 (0.00%)	7 (6.42%)	0.10

AKI, acute kidney injury; BMI, body mass index; LVEF, left ventricular ejection fraction; NYHA, New York Heart Association; CABG, coronary artery bypass graft; CPB, cardiopulmonary bypass; ICU, intensive care unit; RRT, renal replacement therapy; Other aortic diseases include aortic penetrating ulcer, aortic intramural hematoma, and pseudoaneurysm; Combined surgery, procedures that involved at least two sites among the aortic root, ascending aorta, arch, and descending aorta.

### 3.2 The Development and Internal Validation of a Cytokine-Based Predictive Model for AKI after Aortic Surgery

**Fig. 1.**
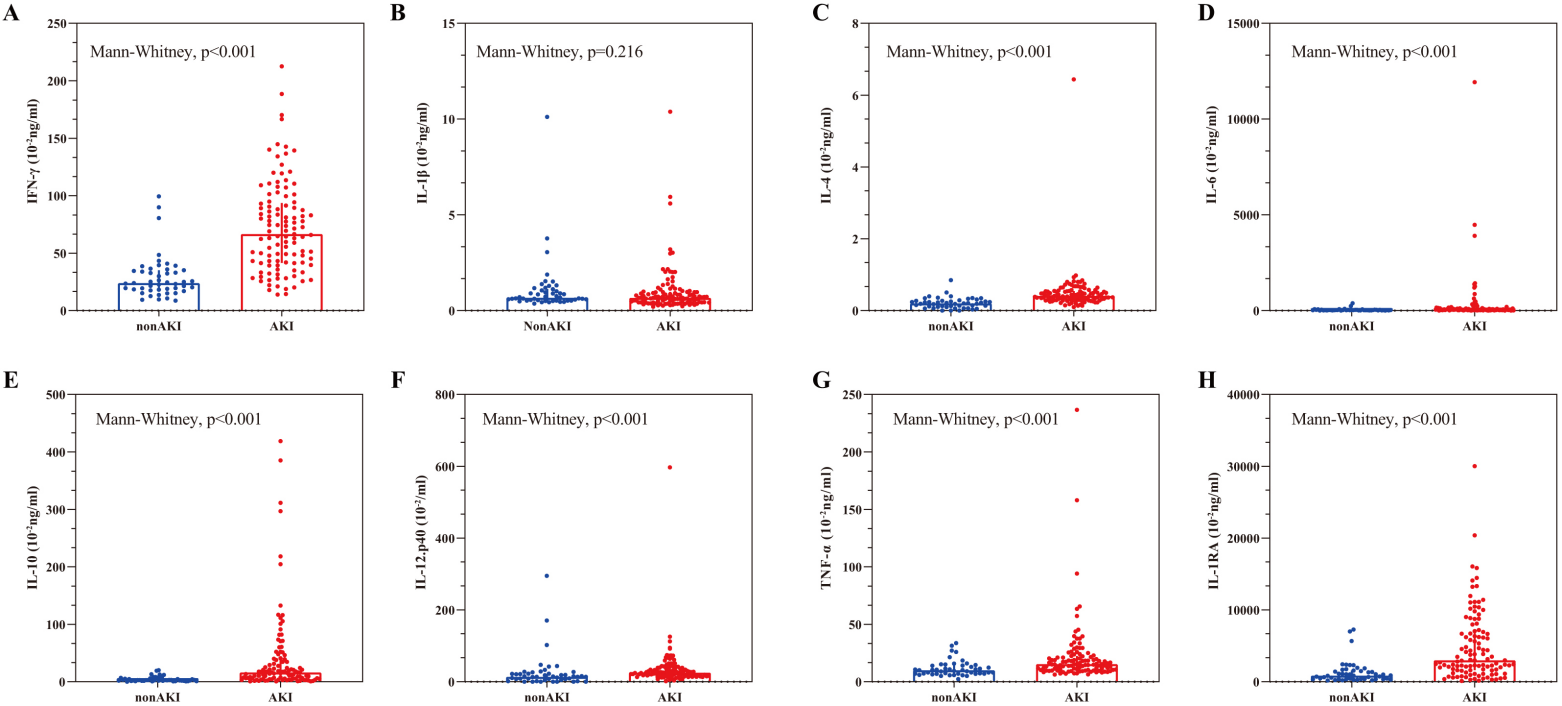
**Comparisons of cytokine concentrations between AKI and non-AKI groups**. (A–H) The figure sequentially presents the concentrations of IFN-γ, IL-1β, IL-4, IL-6, IL-10, IL-12p40, TNF-α, and IL-1RA in both the AKI and non-AKI cohorts. The top of the box shows the median and the vertical bar shows the interquartile range. The red dots indicate patients with AKI, and the blue-green dots indicate patients without AKI. AKI, acute kidney injury; IFN-γ, interferon-gamma; IL-1β, interleukin-1beta; IL-6, interleukin-6; IL-12p40, interleukin-12p40; TNF-α, tumor necrosis factor-alpha; IL-10, interleukin-10; IL-4, interleukin-4; IL-1RA, interleukin-1RA.

To analyze the distinct feature of macrophage polarization-related cytokines between the AKI group and the non-AKI group, we assessed the levels of 8 cytokines in our cohort, comparing the concentrations between the groups. As shown in Fig. 1, the AKI group exhibited significantly higher concentrations of seven of the eight cytokines, with IL-1β being the sole exception. Supporting these findings, univariate logistic regression analysis demonstrated that these seven cytokines (IFN-γ, IL-4, IL-6, IL-10, IL-12p40, IL-1RA, and TNF-α) were significantly associated with AKI development (*p <* 0.05) as shown in Table 2.

**Table 2.** **Univariate regression analysis between 8 cytokines and AKI**.

Factors	OR	95% CI	*p* value
lnIFN-γ	13.55	5.77–31.81	<0.001
lnIL-1β	0.76	0.46–1.25	0.280
lnIL-4	15.89	5.87–43.04	<0.001
lnIL-6	1.92	1.32–2.80	0.001
lnIL-10	2.94	1.97–4.37	<0.001
lnIL-12p40	1.97	1.23–3.16	0.005
lnTNF-α	6.41	2.75–14.94	<0.001
lnIL-1RA	2.81	1.91–4.13	<0.001

lnIFN-γ, natural logarithm transformed IFN-γ; lnIL-1β, natural logarithm transformed IL-1β; lnIL-4, natural logarithm transformed IL-4; lnIL-6, natural logarithm transformed IL-6; lnIL-10, natural logarithm transformed IL-10; lnIL-12p40, natural logarithm transformed IL-12p40; lnTNF-α, natural logarithm transformed TNF-α; InIL-1RA, natural logarithm transformed IL-1RA; IFN-γ, interferon-gamma; IL-4, interleukin-4; IL-1β, interleukin-1beta; IL-6, interleukin-6; IL-10, interleukin-10; IL-12p40, interleukin-12p40; TNF-α, tumor necrosis factor-alpha; IL-1RA, interleukin-1RA; AKI, acute kidney injury; OR, odds ratios; CI, confidence interval.

**Fig. 2.**
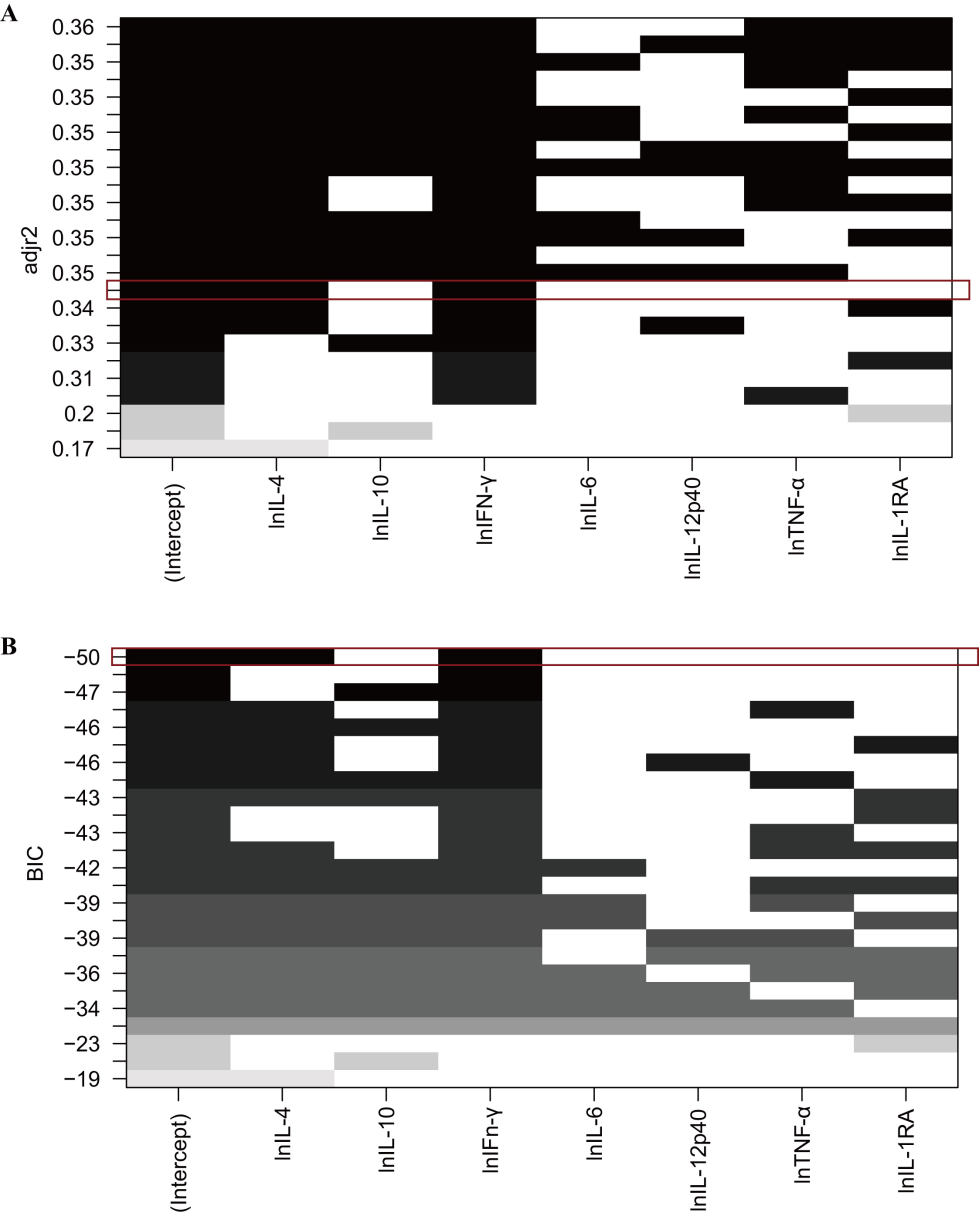
**Model selection using adjusted r-squared value (A) and BIC value (B)**. The empty bars signify variables not included in the models, while the gray and black bars indicate the included variables. (A) Displays the adjusted r-squared values of each model. For example, the red box highlights a model incorporating lnIL-4 and lnIFN-γ with an adjusted r-squared value of 0.35. (B) Shows the BIC values for each model. Here again, the red box underscores a model featuring lnIL-4 and lnIFN-γ with a BIC value of –50. adjr2, adjusted r-squared value; BIC, Bayesian information criterion; lnIL-4, natural logarithm transformed IL-4; lnIL-10, natural logarithm transformed IL-10; lnIFN-γ, natural logarithm transformed IFN-γ; lnIL-6, natural logarithm transformed IL-6; lnIL-12p40, natural logarithm transformed IL-12p40; lnTNF-α, natural logarithm transformed TNF-α; lnIL-1RA, natural logarithm transformed IL-1RA; IL-4, interleukin-4; IL-10, interleukin-10; IFN-γ, interferon-gamma; IL-6, interleukin-6; IL-12p40, interleukin-12p40; TNF-α, tumor necrosis factor-alpha; IL-1RA, interleukin-1RA.

We included these seven cytokines in an all-subsets regression analysis to identify the most predictive model for postoperative AKI. The model performance was evaluated using the adjusted r-squared value and Bayesian Information Criterion (BIC) values as shown in Fig. 2. Although the model including IL-4, IL-10, IFN-γ, TNF-α, and IL-1RA had the highest adjusted r-squared value (0.36), its BIC value was –42. In contrast, the model limited to IL-4 and IFN-γ had a slightly lower adjusted r-squared value of 0.35, but included the lowest BIC value of –50. Given the trade-off between the adjusted r-squared and BIC values, the model including IFN-γ and IL-4 emerged as the optimal cytokine-based predictor. Utilizing these two factors, a logistic regression model was then established to identify patients at higher risk of developing AKI after aortic surgery (Table 3).

**Table 3.** **Parameters of the cytokine-based model**.

Cytokine-based model	Estimate	SE	Z values	*p* value
Intercept	–3.373	1.970	–1.712	0.086
lnIFN-γ	1.871	0.547	3.418	<0.001
lnIL-4	1.829	0.471	3.880	<0.001

SE, standard error; lnIFN-γ, natural logarithm transformed IFN-γ; lnIL-4, natural logarithm transformed IL-4; IFN-γ, interferon-gamma; IL-4, interleukin-4.

**Fig. 3.**
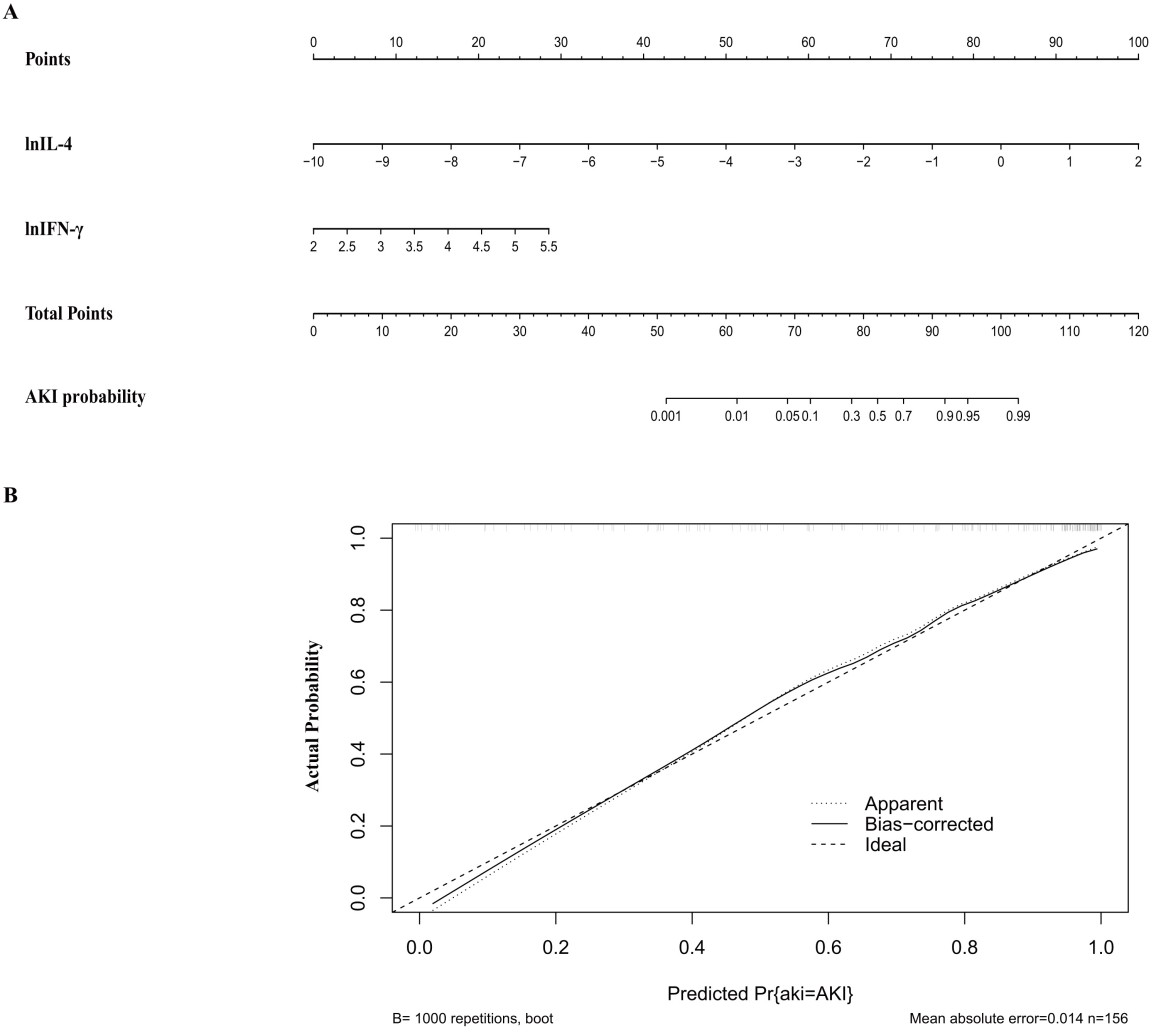
**Nomogram (A) and calibration curve (B) of the cytokine-based model**. (A) Nomogram for cytokine-based AKI prediction. Logarithmic transformation is recommended for IL-4 and IFN-γ concentrations in clinical application. To determine the points for the two cytokines, a line is drawn from the corresponding concentration value to the “Points” line. Find the sum these individual points to determine the total score for each cytokine. By drawing a vertical line from the total points line to the AKI probability line, the probability of AKI can be determined. (B) Calibration curve of the cytokine-based model. lnIL-4, natural logarithm transformed IL-4; lnIFN-γ, natural logarithm transformed IFN-γ; IL-4, interleukin-4; IFN-γ, interferon-gamma; AKI, acute kidney injury.

A nomogram for the prediction of postoperative AKI in patients undergoing aortic surgery was constructed (Fig. 3A). The nomogram and was based on the regression coefficients and the intercept in the model featuring IFN-γ and IL-4. Points were assigned to the early postoperative levels of IFN- and IL-4. Summing these points provides a total score from which the corresponding probability of developing AKI can then be predicted on the bottom axis.

The cytokine-based predictive model was validated internally by the bootstrapping method (1000 resamples). This confirmed the model’s strong discriminative power, reflected by a closely matching corrected C-statistic of 0.89 (Table 4). The calibration curve for the cytokine-based model illustrated a strong congruence between the model’s predicted AKI risk and the actual AKI in the study patients (Fig. 3B).

**Table 4.** **Comparison between the clinical factor-based model and the cytokine-based model**.

Models	AIC	BIC	C-statistics	Corrected C-statistics	Brier	Sensitivity	Specificity	Categorical NRI	IDI
Cytokine-based model	115.75	124.90	0.90 (0.85–0.96)	0.89 (0.84–0.95)	0.11	0.78	0.89	0.43 (0.23–0.63) *p * < 0.001	0.34 (0.23–0.45) *p * < 0.001
Clinical factor-based model	179.48	194.73	0.72 (0.63–0.82)	0.69 (0.60–0.79)	0.18	0.79	0.60	ref	ref

Cytokine-based model: lnIFN-γ + lnIL-4; Clinical factor-based model: age + hypertension + preoperative serum creatinine + prolonged cardiopulmonary bypass time (>120 min); lnIFN-γ, natural logarithm transformed IFN-γ; lnIL-4, natural logarithm transformed IL-4; IFN-γ, interferon-gamma; IL-4, interleukin-4; AIC, akaike information criterion; BIC, Bayesian Information Criterion; Corrected C statistics, bias correction based on 1000 internal replications by bootstrapping; NRI, net reclassification improvement; IDI, integrated discrimination improvement; ref, reference.

### 3.3 Comparison between the Cytokine-Based Model and the Clinical Factor-Based Model

Previous studies [22, 23, 24, 25, 26] have found associations between clinical variables such as age, history of hypertension, preoperative blood creatinine, prolonged CPB time, and elevated AKI risk of AKI following aortic surgery. We next compared the predictive ability of these clinical factor-based models was to our cytokine-based model (Table 4).

**Fig. 4.**
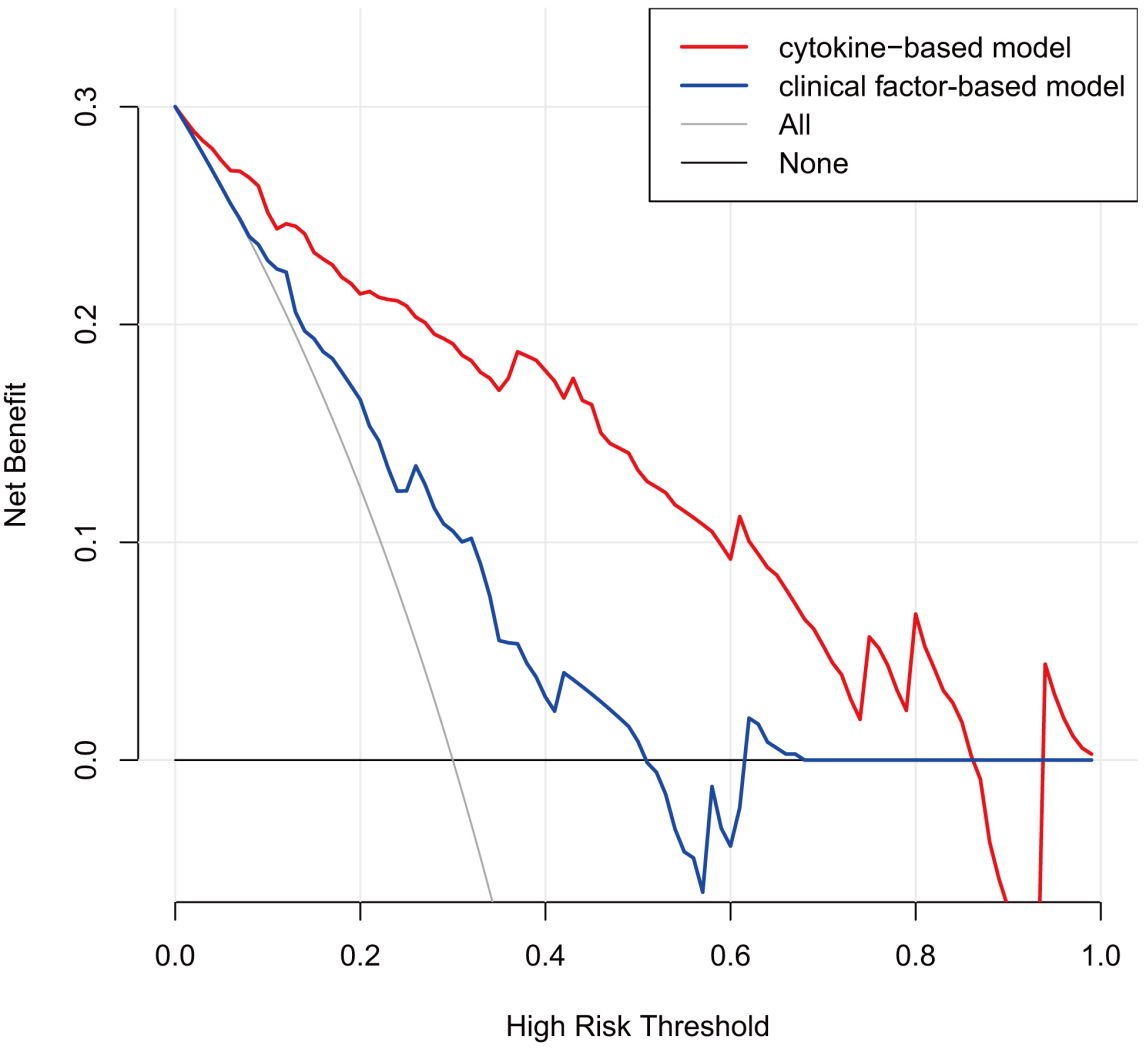
**Decision curve analysis for comparing the cytokine-based model and clinical factor-based model**. Cytokine-based model: lnIFN-γ + lnIL-4; Clinical factor-based model: age + hypertension + preoperative serum creatinine + prolonged cardiopulmonary bypass time (>120 min); lnIFN-γ, natural logarithm transformed IFN-γ; lnIL-4, natural logarithm transformed IL-4; IFN-γ, interferon-gamma; IL-4, interleukin-4.

Compared to traditional clinical factor-based models for predicting postoperative AKI, our cytokine-based model demonstrated superior performance across multiple evaluation metrics. Specifically, the cytokine-based model has a superior goodness of fit score (akaike information criterion [AIC]: 115.75 vs. 179.48; BIC: 124.90 vs. 194.73). Furthermore, the model has improved calibration, with a Brier score of 0.11 compared to 0. 18 for the clinical model. The discrimination performance was also improved, yielding a C-statistic of 0.90 (95% confidence interval [CI]: 0.85–0.96) vs. 0.72 (95% CI: 0.63–0.82). Notably, the cytokine-based approach significantly enhanced patient reclassification, with a net reclassification improvement (NRI) of 0.43 (95% CI: 0.23–0.63, *p* < 0.001), and integrated discrimination improvement (IDI of 0.34 (95% CI: 0.23–0.45, *p* < 0.001). In addition, the cytokine-based model outperformed the clinical factor-based model in terms of net benefit, as shown by the decision curve analysis (Fig. 4).

### 3.4 The Predictive Performance of the Cytokine-Based Model in Stratified Groups by Age/Sex/Type of Surgery

We assessed the generalizability of our cytokine-based AKI prediction model, partly due to the male-dominated gender distribution, wide age, and surgical complexity. The cytokine-based model was used to predict AKI across a range of age, gender, and surgical type groups (Table 5). The model showed robust predictive power in both genders, albeit with slightly superior discrimination in females (AUC: 0.96 vs. 0.88; Brier: 0.08 vs. 0.11). Similarly, the cytokine-based model performed well in both the older group (age ≥60 years) and the younger group (age <60 years), with relatively better discrimination in older patients (AUC: 0.91 vs. 0.89; Brier: 0.09 vs. 0.12). Since there were relatively few patients in the study population who underwent simple aortic arch surgery and simple descending aortic surgery, we grouped them with patients who underwent simple aortic root surgery for ease of analysis. This model also showed good performance in the aortic root/aortic arch/descending aorta surgery groups and the combined surgery group.

**Table 5.** **Performance of the cytokine-based model in age/sex/surgery type groups**.

Groups	AKI/non-AKI	AUC	Brier
Male group	84/34	0.88	0.11
Female group	25/13	0.96	0.08
Age ≥60 years group	44/19	0.91	0.09
Age <60 years group	65/28	0.89	0.12
Aortic root/aortic arch/descending aorta surgery group	40/34	0.92	0.01
Combined surgery group	69/13	0.89	0.08

AUC, the area under the receiver operator characteristic curve; AKI, acute kidney injury; Combined surgery, procedures involving at least two sites among the aortic root, ascending aorta, arch, and descending aorta.

### 3.5 Discrimination of Selected Cytokines for AKI and Composite Outcomes

**Fig. 5.**
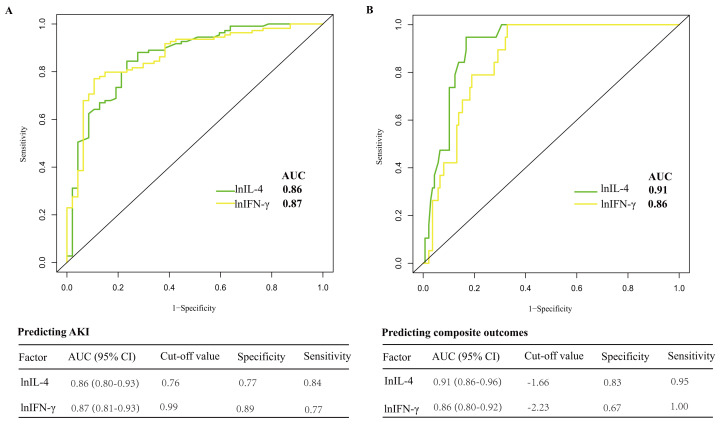
**Discriminability of selected cytokines for AKI (A) and composite outcomes (B)**. (A) In predicting AKI, the AUC for lnIL-4 was 0.86, with a specificity of 0.77 and a sensitivity of 0.84; the AUC for lnIFN-γ was 0.87, with a specificity of 0.89 and a sensitivity of 0.77. (B) In predicting composite outcomes including renal replacement treatment and in-hospital death, the AUC for lnIL-4 was 0.91, with a specificity of 0.83 and sensitivity of 0.95; the AUC for lnIFN-γ was 0.86, with a specificity of 0.67 and sensitivity of 1.00. AUC, area under the curve; lnIFN-γ, natural logarithm transformed IFN-γ; lnIL-4, natural logarithm transformed IL-4; IFN-γ, interferon-gamma; IL-4, interleukin-4; AKI, acute kidney injury.

**Fig. 6.**
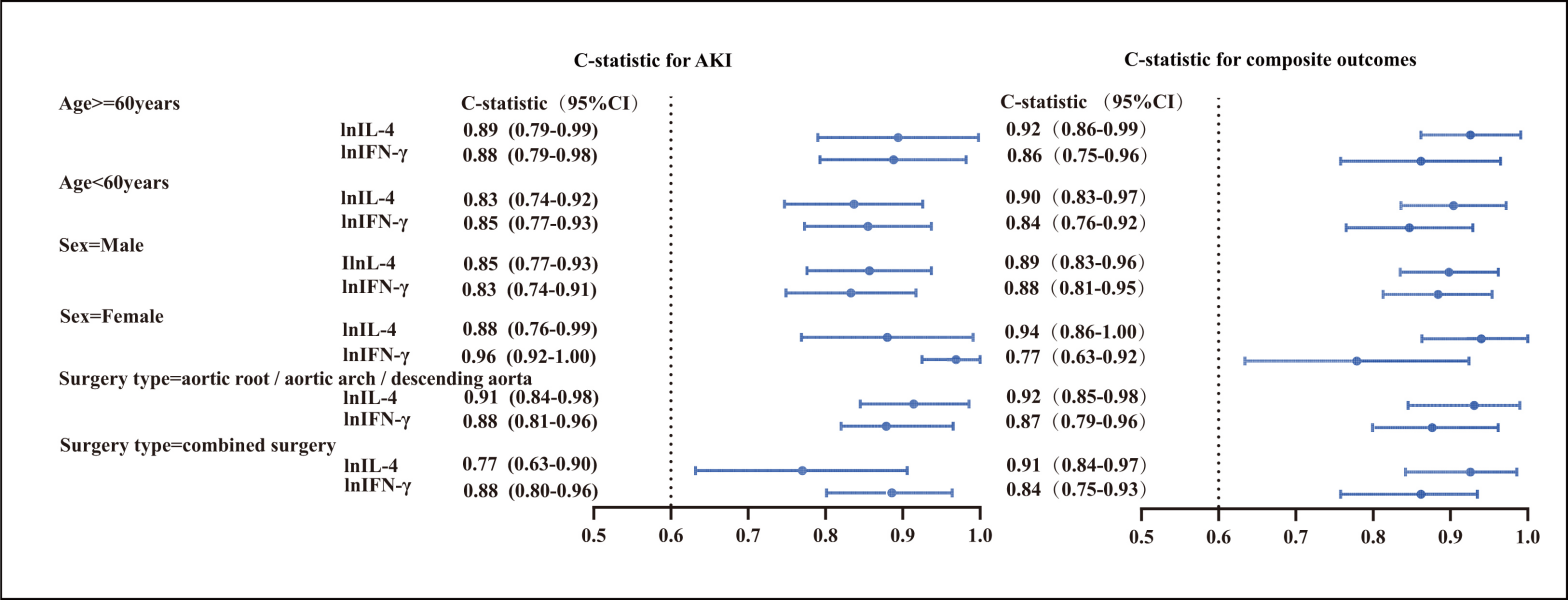
**Discriminability of selected cytokines for AKI and composite outcomes in stratified groups based on age, gender, and surgery type**. C-statistics and 95% CIs of cytokines (IFN-γ and IL-4) for predicting AKI and composite outcome in different subgroups were shown on the left and right side, respectively. AKI, acute kidney injury; lnIFN-γ, natural logarithm transformed IFN-γ; lnIL-4, natural logarithm transformed IL-4; IFN-γ, interferon-gamma; IL-4, interleukin-4; CI, confidence interval.

We evaluated the individual discriminative ability of IL-4 and IFN-γ for predicting AKI and composite outcomes from the predictive model by receiver operating characteristics analysis. As shown in Fig. 5, IL-4 and IFN-γ not only demonstrated a strong ability to discriminate AKI with AUCs of 0.86 and 0.87, respectively but also had good discriminability for composite outcomes with AUCs of 0.91 and 0.86, respectively. In addition, the discriminability of IL-4 and IFN-γ was also good in the groups stratified by age/sex/type of surgery (Fig. 6).

### 3.6 Association of Selected Cytokines with AKI and Hospital Outcomes

We further evaluated the performance of the 2 cytokines in stratifying postoperative AKI risk. As presented in Table 6, elevated concentrations of IFN-γ and IL-4 (stratified by their tertiles) were strongly predictive of elevated AKI risk. Importantly, these associations held true even after adjusting for age, sex, and other relevant confounding variables.

**Table 6.** **Association of selected cytokines with AKI**.

Cytokines	Crude odds ratios (95% CI)	*p* value	Adjusted I odds ratios (95% CI)	*p* value	Adjusted II odds ratios (95% CI)	*p* value
IFN-γ group						
Tertile 1	ref		ref		ref	
8.70–33.23 (${\mathrm{10}}^{–\,2}$ ng/mL)						
Tertile 2	6.47 (2.71–15.49)	<0.001	6.71 (2.74–16.41)	<0.001	12.39 (3.63–42.20)	<0.001
32.23–74.25 (${\mathrm{10}}^{–\,2}$ ng/mL)						
Tertile 3	28.37 (7.77–103.59)	<0.001	32.10 (8.47–121.60)	<0.001	55.32 (10.65–287.33)	<0.001
74.25–212.58 (${\mathrm{10}}^{–\,2}$ ng/mL)						
*p* for group trend		<0.001		<0.001		<0.001
IL-4 group						
Tertile 1	ref		ref		ref	
0–0.27 (${\mathrm{10}}^{–\,2}$ ng/mL)						
Tertile 2	7.46 (3.71–18.05)	<0.001	7.21 (2.96–17.53)	<0.001	5.32 (1.85–15.31)	0.002
0.27–0.42 (${\mathrm{10}}^{–\,2}$ ng/mL)						
Tertile 3	51.00 (11.07–235.07)	<0.001	54.44 (11.56–256.38)	<0.001	68.05 (10.80–428.62)	<0.001
0.42–6.44 (${\mathrm{10}}^{–\,2}$ ng/mL)						
*p* for group trend		<0.001		<0.001		<0.001

Adjusted I: adjusted for age and sex. Adjusted II: adjusted for age, sex, BMI, preoperative serum creatinine, hypertension, types of surgery, prolonged cardiopulmonary bypass time, NYHAIII-IV. IFN-γ, interferon-gamma; IL-4, interleukin-4; AKI, acute kidney injury; Tertile 1, first tertile; Tertile 2, second tertile; Tertile 3, third tertile; BMI, body mass index; NYHA, New York Heart Association; ref, reference.

Specifically, compared to patients in the lowest tertile of cytokine levels, those in the upper tertiles had markedly increased adjusted odds ratios for developing AKI. For IFN-γ, the adjusted odds ratio escalated from 12.29 in the second tertile to 55.32 in the third tertile, both with p-values below 0.001. Similarly, for IL-4, the adjusted odds ratio climbed from 5.32 in the second tertile to 68.05 in the third tertile, also registering *p*-values below 0.001. This reveals a strong and graded association between elevated cytokine levels and heightened risk of postoperative AKI.

**Fig. 7.**
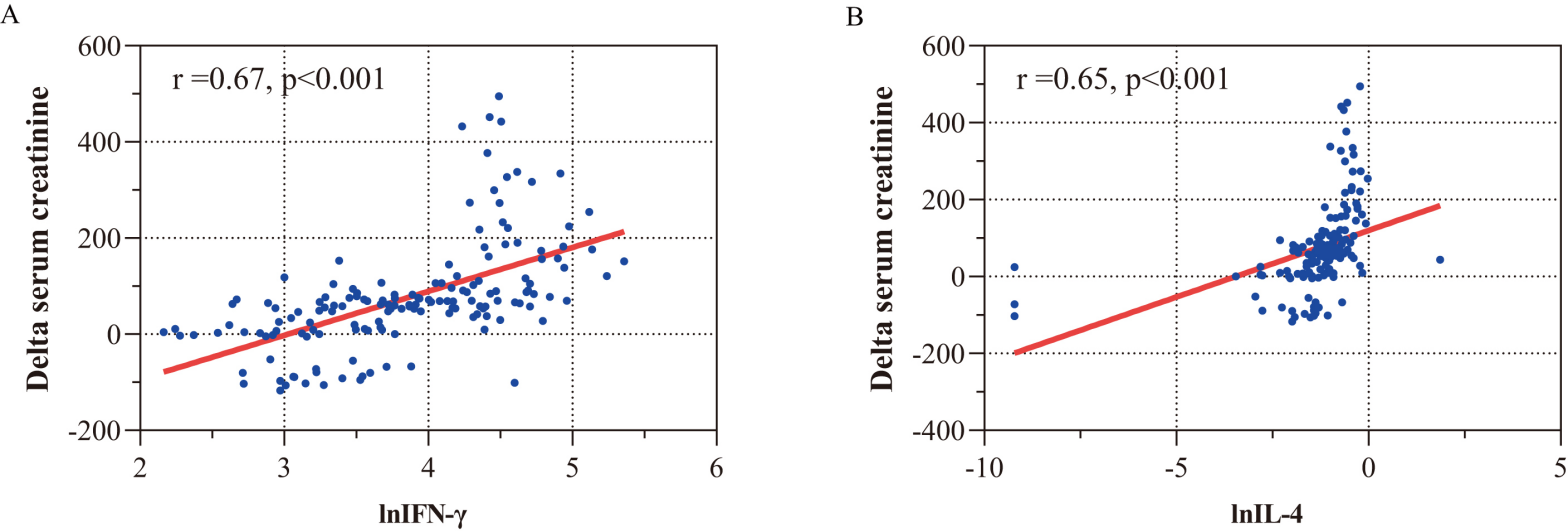
**Correlations between IFN-γ (A), IL-4 (B), and alteration of creatinine**. (A) Correlation between IFN-γ and delta creatinine. (B) Correlation between IL-4 and delta creatinine. Delta serum creatinine, change in serum creatinine after aortic surgery versus preoperative creatinine. lnIFN-γ, natural logarithm transformed IFN-γ; lnIL-4, natural logarithm transformed IL-4; IFN-γ, interferon-gamma; IL-4, interleukin-4; The concentrations of cytokines in the graph were ln transformed. The Spearman correlation coefficient is shown.

Additionally, the concentrations of IFN-γ and IL-4 were highly correlated with changes in serum creatinine, as shown by the Spearman correlation test (IFN-γ, r = 0.67; IL-4, r = 0.65) (Fig. 7). This suggests that both IFN-γ and IL-4 may play a role in determining the severity of AKI. Therefore, we also assessed the performance of these two cytokines in stratifying the risk of severe AKI and composite outcomes, as well as associated outcomes. As shown in Table 7, patients with IFN-γ or IL-4 in the second and third tertile were more likely to develop severe AKI (AKIN stage 2/3) and experience composite outcomes compared to those in the first tertile (all *p* for group trend <0.001). Furthermore, increasing levels of IFN-γ and IL-4 were associated with significantly extended ICU stays and longer durations of mechanical ventilation (all *p* for group trend <0.001). Although patients with IFN-γ or IL-4 in the upper tertile had longer hospital stays than those in the first tertile, the differences were not statistically significant (*p* for group trend: IFN-γ, 0.608; IL-4, 0.238).

**Table 7.** **Association of selected cytokines with in-hospital outcomes**.

Cytokine	Tertiles	Hospitalization time (days)	ICU stay (days)	Ventilation time (hours)	AKIN	AKIN	Composite outcomes
		(median (IQR))	(median (IQR))	(median (IQR))	stage 2	stage 3	(n (%))
IFN-γ (${\mathrm{10}}^{–\,2}$ ng/mL)	Tertile 1 (8.70–33.23)	14.00 (11.25–22.75)	2.00 (2.00–4.00)	16.00 (11.00–24.50)	3 (5.77%)	1 (1.92%)	0 (0.00%)
	Tertile 2 (33.23–74.25)	15.50 (12.00–23.00)	3.00 (2.00–6.00)	18.00 (15.75–28.75)	6 (11.54%)	3 (5.77%)	4 (7.69%)
	Tertile 3 (74.25–212.58)	18.00 (11.25–26.50)	6.00 (4.00–12.00)	52.00 (24.00–156.00)	12 (23.08%)	21 (40.38%)	15 (28.85%)
	*p* value for group trend	0.608	<0.001	<0.001	<0.001	<0.001	<0.001
IL-4 (${\mathrm{10}}^{–\,2}$ ng/mL)	Tertile 1 (0–0.27)	14.00 (11.00–22.00)	3.00 (2.00–4.00)	17.00 (15.25–29.50)	4 (7.84%)	0 (0.00%)	0 (0.00%)
	Tertile 2 (0.27–0.42)	14.50 (11.25–25.00)	4.00 (2.00–5.25)	19.50 (14.00–36.25)	6 (11.54%)	3 (5.77%)	1 (1.92%)
	Tertile 3 (0.42–6.44)	18.00 (13.00–24.00)	5.50 (2.25–13.00)	35.00 (18.25–139.25)	11 (20.75%)	22 (41.51%)	18 (33.96%)
	*p* value for group trend	0.238	<0.001	<0.001	<0.001	<0.001	<0.001

Composite outcome, renal replacement therapy and/or in-hospital death; IQR, interquartile range; IFN-γ, interferon-gamma; IL-4, interleukin-4; Tertile 1, first tertile; Tertile 2, second tertile; Tertile 3, third tertile; ICU, intensive care unit; AKIN, acute kidney injury network.

## 4. Discussion

Our study found that elevated levels of cytokines related to macrophage polarization, specifically IFN-γ and IL-4, are significantly associates with AKI development in patients undergoing CPB surgery. The cytokines IFN-γ and IL-4 were selected as predictors of AKI by our all-subset regression model. Compared to models reliant on clinical factors like age, hypertension, preoperative serum creatinine, and extended CPB time, our cytokine-based model demonstrated superior predictive accuracy and net clinical benefit for AKI. Furthermore, both IFN-γ and IL-4 appear to function as independent risk factors for AKI, with higher levels of each cytokine correlating with more severe AKI and adverse composite outcomes.

AKI is a frequent complication following aortic surgery and is strongly linked to poor clinical outcomes, including the need for RRT and increased mortality. Current diagnostic measures for AKI, including serum creatinine levels, fall short in early detection of renal tubular injury due to their delayed elevation and low sensitivity [27]. A study involving 303 patients was designed to assess the correlation between AKI (as defined by serum creatinine) and diffuse histologic criteria for acute kidney injury based on renal biopsy. Remarkably, approximately one-third of patients meeting the histological AKI criteria failed to meet AKI diagnostic criteria. This was primarily because serum creatinine did not rise quickly enough to fulfill the definition of AKI [28].

Therefore, there is a considerable need for a reliable biomarker to predict early AKI, as timely intervention can lead to the prevention of multiple clinical complications. In recent years, several novel biomarkers for AKI have been evaluated in aortic surgery. The most studied biomarkers, derived from urine or plasma samples, are related to cell cycle arrest, such as NGAL, liver-type fatty acid-binding protein (L-FABP), IGFBP-7, TIMP-2, and cystatin C [7, 8, 9, 10, 11, 29, 30, 31, 32]. A few studies have evaluated the predictive ability of blood immune-inflammatory biomarkers such as plasma secretory leukocyte peptidase inhibitor (SLPI), pentraxin 3 (PTX3), IL1-RA, MCP-1, suppressor of tumorigenicity 2 (ST-2), IL-6, and IL-10 [19, 20, 33, 34]. However, these heterogeneous studies mainly evaluated the association between an individual or multiple selected cytokines and AKI based on a small number of samples and most were limited to a specific setting, with wide variations in predictive performance. As a result, it has been suggested that these studies may have introduced a selection bias that has undermined the ability of individual biomarkers to predict AKI.

Our study differs from previous research by aiming to develop and validate a novel robust predictive model specifically for detecting AKI in patients who underwent aortic surgery with CPB. We evaluated eight macrophage polarization-related cytokines (including IFN-γ, IL-4, IL-6, IL-10, IL-1RA, IL-1β, IL-12p40, and TNF-α) in a cohort of 156 patients undergoing a diverse range of aortic surgery. As shown in Fig. 1, the Mann-Whitney analysis showed the concentrations of IFN-γ, IL-4, IL-6, IL-10, IL-1RA, IL-12p40 and TNF-α were significantly higher in the AKI group compared with the non-AKI group (*p* < 0.001). In contrast, there was no difference in IL-1β levels between the AKI and non-AKI groups (*p* = 0.216). Furthermore, univariate regression analysis also showed that these 7 cytokines (IFN-γ, IL-4, IL-6, IL-10, IL-1RA, IL-12p40 and TNF-α) were significantly associated with AKI (*p* < 0.05), whereas IL-1β was not (*p* = 0.280) (Table 2). These 7 cytokines showed statistical significance between the AKI and non-AKI groups in univariate analysis, indicating that multiple macrophage polarization-related cytokines, rather than 1 or 2 cytokines, are involved in postoperative AKI.

To objectively select the most predictive combination of cytokines for estimating AKI among these 7 cytokines, we used all-subset regression, also known as best subset selection. It can fit all possible combination models of predictive variables and then select the best model under the condition of the existing variables according to the adjusted r-squared and BIC. The adjusted r-squared value reflects the explanatory power of the combination of predictors on the dependent variable, while the BIC value reflects the goodness of fit of the model. Given the high adjusted r-squared and the lowest BIC, IFN-γ and IL-4 were selected to build the predictive model. Binary logistic regression was used to build the predictive model. The predictive model based on IFN-γ and IL-4 yielded a powerful discriminative ability of AKI. Even after separating the entire population into subgroups based on age, sex, and type of surgery, positive results were still obtained from this model. Our model outperformed the standard clinical factor-based models in multiple subgroups including females, the elderly, and patients with a single surgical site, reflecting improved clinical applicability and reliability.

In clinical practice, the standard models for predicting postoperative AKI typically rely on patient clinical data. Several clinical characteristics of patients, such as age, preoperative serum creatinine, history of hypertension, poor preoperative cardiac functional status, and prolonged CPB time, have been considered risk factors for AKI after aortic surgery [22, 23, 24, 25, 26]. Zhang and his colleagues reported 4 clinical prediction models for AKI based on perioperative variables for patients with acute type A aortic dissection (ATAAD) undergoing Sun’s procedure, with the AUC ranging from 0.710 to 0.848 [35]. Kim and his colleagues [36] reported a simplified clinical score including 6 clinical variables (age, preoperative glomerular filtration rate, left ventricular ejection fraction, operation time, intraoperative urine output, and intraoperative use of furosemide) to stratify the risk of postoperative AKI in patients undergoing aortic surgery, with an AUC of 0.740. Although the variables of these prediction models were easy to collect, most of them lacked specificity, and their predictive power may largely depend on the richness of the variables. In our study, we compared a standard clinical factor-based prediction model using age, sex, hypertension history, NYHA III-IV, and prolonged CPB time to our cytokine-based prediction model described above. Consistent with the existing literature, our clinical factor-based model had an average predictive power with an AUC of 0.72, which was much lower than our cytokine-based prediction model with an AUC of 0.90. In addition, our cytokine model had many advantages over the clinical factor model in terms of patient reclassification and net clinical benefit, as well as model parsimony.

To the best of our knowledge, while both IFN-y and IL-4 are known to play contribute towards AKI pathophysiology, their predictive value in post-surgical AKI has not been previously documented in the existing literature. IFN-γ, the typical cytokine produced by classically activated macrophages (M1), has been implicated in CD4+ T cell-mediated ischemia-reperfusion injury [37]. While derived from alternatively activated macrophages (M2), IL-4 has been involved in tissue repair and recovery from ischemia-reperfusion injury and promoted renal tubular interstitial fibrosis in animal models [38, 39]. In a study by Moledina and colleagues, both IFN-γ and IL-4 were elevated in the blood within 6 hours of surgery and were independently associated with both AKI and decreased one-year mortality rates following cardiac surgery [40]. Similarly, our study demonstrates for the first time that IFN-γ and IL-4 were significantly increased by the first day following surgery and were independently associated with AKI after aortic surgery. Specifically, after adjustment for confounders, the second and third tertiles of IL-4 had a 5.32- and 68.05-fold greater risk of AKI, respectively, than the first tertile. In contrast, the risk of AKI in the second and third tertiles of IFN-γ was 12.39-fold and 55.32-fold higher, than in the first tertile. In addition, we found that patients in the highest tertiles of IFN-γ or IL-4 were at high risk for severe AKI, composite outcomes, longer ICU stays, longer hospital stays, and longer periods of mechanical ventilation. Furthermore, each cytokine had prognostic value for the prediction of increased risk of AKI and composite outcomes across age, sex, and surgery type groups. In conclusion, our results suggest that IL-4 and IFN-γ not only independently predicted AKI after aortic surgery, but also increased morbidity and mortality in these patients. Thus, IL-4 and IFN-γ are promising potential therapeutic targets for the treatment of AKI. Further large-scale studies are needed to confirm these findings in the future.

Since IFN-γ and IL-4 were significantly associated with postoperative AKI in both adult aortic surgery and adult cardiac surgery, macrophage polarization-related cytokines may represent a common pathophysiological mechanism involved in postoperative AKI with CPB. Given the encouraging results of our study, we look forward to extending our study to patients undergoing cardiac surgery with CPB to predict AKI in the future.

There were some notable differences between our study and other studies reported in the literature on this subject. For example, the serum concentrations of IFN-γ were significantly higher in patients with AKI after surgery in our study. However, in a study of the repair of congenital cardiac defects with CPB, children with AKI did not have significantly elevated serum IFN-γ levels at 2, 12, and 24 hours after CPB compared with patients who did not develop AKI [41]. In addition, IFN-γ and IL-4 levels strongly correlated with AKI severity in our study, whereas children with progressive AKI did not show a significant difference in IFN-γ and IL-4 levels compared to children without progressive AKI after surgery in another cohort of patients with congenital heart disease [42]. These differences suggest that the immunoinflammatory process in postoperative AKI is complex and may differ between study populations and types of surgery. This may be due to the differences in the pathophysiology between children and adults, as well as the differences in operations between adult aortic and congenital heart surgery, that may influence the immunoinflammatory pathways that affect the various changes in blood cytokines. Additionally, cytokine concentrations may differ based on the time of sampling, sample source, and methods of measurement. Therefore, when employing these cytokines or models for the prediction of AKI in clinical practice, it is necessary to consider the conditions of their application, such as the appropriate objectives, specific types of surgery, the time of sampling, the sample source, and the methods of measurement.

## 5. Limitations

This study has multiple limitations warranting discussion. First, its retrospective, observational, and single center nature potentially introduces selection bias, rendering the findings less generalizable. Since some selection bias may be inevitable, the results cannot be considered conclusive. Second, while we conducted internal validation using 1000 bootstrap samples, external validation from a broader array of hospitals is required to confirm the model’s robustness. Third, this study had a limited sample. Since the criteria for AKI are not uniform, we could have missed some patients who had subsequently developed AKI had we followed the Acute Kidney Injury Network guideline, which only takes into account a blood creatinine rise within 48 hours following surgery. Finally, our predictive model is limited because it is based on cytokines, whose assays may not be available in some hospitals.

## 6. Conclusions

In patients undergoing aortic surgery with CPB, we found a strong association between macrophage polarization-related cytokines and postoperative AKI. Our internally validated model, incorporating IFN-γ and IL-4, reliably predicts AKI risks and facilitates AKI risk stratification in patients undergoing aortic surgery. Patients with high levels of IFN-γ and IL-4 were more likely to experience delayed recovery, severe AKI, and adverse composite outcomes. Although promising, the model requires external validation from multi-center data for broader clinical application. Once confirmed, this predictive tool could offer clinicians valuable insights for early identification of patients at high risk for AKI development.

## Data Availability

The datasets used and/or analyzed during the current study are available from the corresponding author upon reasonable request.

## Abbreviations

AKI, acute kidney injury; AIC, akaike information criterion; AKIN, acute kidney injury network; BIC, Bayesian information criterion; CPB, cardiopulmonary bypass; DCA, decision curve analysis; ICU, intensive care unit; IDI, integrated discrimination improvement; NGAL, neutrophil gelatinase-associated lipocalin; IGFBP-7, insulin-like growth factor binding protein-7; TIMP-2, tissue inhibitor of metalloproteinase-2; GFR, glomerular filtration rate; LVEF, left ventricular ejection fraction; NRI, net reclassification improvement; ROC, receiver operator characteristic curve; AUC, area under the curve; RRT, renal replacement therapy; IFN-γ, interferon-gamma; IL-4, interleukin-4; MCP-1, monocyte chemotactic protein-1; IL-10, interleukin-10; IL-6, interleukin-6; IL-1RA, interleukin-1 receptor antagonist; BMI, body mass index; IL-1β, interleukin-1β; IL-12p40, interleukin-12p40; TNF-α, tumor necrosis factor-alpha; SLPI, serum leukocyte peptidase inhibitor; PTX3, pentraxin 3; ST-2, suppressor of tumorigenicity-2.

## Supplementary Material

Supplementary material associated with this article can be found, in the online version, at https://doi.org/10.31083/j.rcm2502054.

## References

[1] Nadim MK, Forni LG, Bihorac A, Hobson C, Koyner JL, Shaw A (2018). Cardiac and Vascular Surgery-Associated Acute Kidney Injury: The 20th International Consensus Conference of the ADQI (Acute Disease Quality Initiative) Group. *Journal of the American Heart Association*.

[2] Hobson CE, Yavas S, Segal MS, Schold JD, Tribble CG, Layon AJ (2009). Acute kidney injury is associated with increased long-term mortality after cardiothoracic surgery. *Circulation*.

[3] Meng W, Li R, E L, Zha N (2021). Postoperative acute kidney injury and early and long-term mortality in acute aortic dissection patients: A meta-analysis. *Medicine*.

[4] Wu I, Parikh CR (2008). Screening for kidney diseases: older measures versus novel biomarkers. *Clinical Journal of the American Society of Nephrology*.

[5] Waikar SS, Betensky RA, Emerson SC, Bonventre JV (2012). Imperfect gold standards for kidney injury biomarker evaluation. *Journal of the American Society of Nephrology*.

[6] Kashani K, Al-Khafaji A, Ardiles T, Artigas A, Bagshaw SM, Bell M (2013). Discovery and validation of cell cycle arrest biomarkers in human acute kidney injury. *Critical Care*.

[7] Ueta K, Watanabe M, Iguchi N, Uchiyama A, Shirakawa Y, Kuratani T (2014). Early prediction of acute kidney injury biomarkers after endovascular stent graft repair of aortic aneurysm: a prospective observational study. *Journal of Intensive Care*.

[8] Gombert A, Prior I, Martin L, Grommes J, Barbati ME, Foldenauer AC (2018). Urine neutrophil gelatinase-associated lipocalin predicts outcome and renal failure in open and endovascular thoracic abdominal aortic aneurysm surgery. *Scientific Reports*.

[9] Naruse H, Ishii J, Takahashi H, Kitagawa F, Nishimura H, Kawai H (2018). Predicting acute kidney injury using urinary liver-type fatty-acid binding protein and serum N-terminal pro-B-type natriuretic peptide levels in patients treated at medical cardiac intensive care units. *Critical Care*.

[10] Gombert A, Kotelis D, Rückbeil MV, Barbati M, Martin L, Marx G (2021). Increase of urinary TIMP-2 and IGFBP7 as potential predictor of acute kidney injury requiring renal replacement therapy and patients’ outcome following complex endovascular and open thoracic abdominal aortic aneurysm surgery - a prospective observational study. *VASA. Zeitschrift fur Gefasskrankheiten*.

[11] Waskowski J, Pfortmueller CA, Schenk N, Buehlmann R, Schmidli J, Erdoes G (2021). (TIMP2) x (IGFBP7) as early renal biomarker for the prediction of acute kidney injury in aortic surgery (TIGER). A single center observational study. *PLoS ONE*.

[12] Huen SC, Cantley LG (2017). Macrophages in Renal Injury and Repair. *Annual Review of Physiology*.

[13] Han HI, Skvarca LB, Espiritu EB, Davidson AJ, Hukriede NA (2019). The role of macrophages during acute kidney injury: destruction and repair. *Pediatric Nephrology*.

[14] Xie X, Yang X, Wu J, Ma J, Wei W, Fei X (2020). Trib1 Contributes to Recovery From Ischemia/Reperfusion-Induced Acute Kidney Injury by Regulating the Polarization of Renal Macrophages. *Frontiers in Immunology*.

[15] Sasaki K, Terker AS, Pan Y, Li Z, Cao S, Wang Y (2021). Deletion of Myeloid Interferon Regulatory Factor 4 (Irf4) in Mouse Model Protects against Kidney Fibrosis after Ischemic Injury by Decreased Macrophage Recruitment and Activation. *Journal of the American Society of Nephrology*.

[16] Lech M, Gröbmayr R, Ryu M, Lorenz G, Hartter I, Mulay SR (2014). Macrophage phenotype controls long-term AKI outcomes–kidney regeneration versus atrophy. *Journal of the American Society of Nephrology*.

[17] Nathan CF, Murray HW, Wiebe ME, Rubin BY (1983). Identification of interferon-gamma as the lymphokine that activates human macrophage oxidative metabolism and antimicrobial activity. *The Journal of Experimental Medicine*.

[18] Stein M, Keshav S, Harris N, Gordon S (1992). Interleukin 4 potently enhances murine macrophage mannose receptor activity: a marker of alternative immunologic macrophage activation. *The Journal of Experimental Medicine*.

[19] Brinkman R, HayGlass KT, Mutch WAC, Funk DJ (2015). Acute Kidney Injury in Patients Undergoing Open Abdominal Aortic Aneurysm Repair: A Pilot Observational Trial. *Journal of Cardiothoracic and Vascular Anesthesia*.

[20] Chen X, Zhou J, Fang M, Yang J, Wang X, Wang S (2022). Procalcitonin, Interleukin-6 and C-reactive Protein Levels Predict Renal Adverse Outcomes and Mortality in Patients with Acute Type A Aortic Dissection. *Frontiers in Surgery*.

[21] Mehta RL, Kellum JA, Shah SV, Molitoris BA, Ronco C, Warnock DG (2007). Acute Kidney Injury Network: report of an initiative to improve outcomes in acute kidney injury. *Critical Care*.

[22] Nota H, Asai T, Suzuki T, Kinoshita T, Ikegami H, Takashima N (2014). Risk factors for acute kidney injury in aortic arch surgery with selective cerebral perfusion and mild hypothermic lower body circulatory arrest. *Interactive Cardiovascular and Thoracic Surgery*.

[23] Zhou H, Wang G, Yang L, Shi S, Li J, Wang M (2018). Acute Kidney Injury After Total Arch Replacement Combined With Frozen Elephant Trunk Implantation: Incidence, Risk Factors, and Outcome. *Journal of Cardiothoracic and Vascular Anesthesia*.

[24] Xu S, Liu J, Li L, Wu Z, Li J, Liu Y (2019). Cardiopulmonary bypass time is an independent risk factor for acute kidney injury in emergent thoracic aortic surgery: a retrospective cohort study. *Journal of Cardiothoracic Surgery*.

[25] Novak Z, Zaky A, Spangler EL, McFarland GE, Tolwani A, Beck AW (2021). Incidence and predictors of early and delayed renal function decline after aortic aneurysm repair in the Vascular Quality Initiative database. *Journal of Vascular Surgery*.

[26] Chen P, Chen M, Chen L, Ding R, Chen Z, Wang L (2022). Risk factors for severe acute kidney injury post complication after total arch replacement combined with frozen elephant trunk, in acute type A aortic dissection. *Cardiovascular Diagnosis and Therapy*.

[27] Vanmassenhove J, Kielstein J, Jörres A, Biesen WV (2017). Management of patients at risk of acute kidney injury. *The Lancet*.

[28] Chu R, Li C, Wang S, Zou W, Liu G, Yang L (2014). Assessment of KDIGO definitions in patients with histopathologic evidence of acute renal disease. *Clinical Journal of the American Society of Nephrology*.

[29] Obata Y, Kamijo-Ikemori A, Ichikawa D, Sugaya T, Kimura K, Shibagaki Y (2016). Clinical usefulness of urinary liver-type fatty-acid-binding protein as a perioperative marker of acute kidney injury in patients undergoing endovascular or open-abdominal aortic aneurysm repair. *Journal of Anesthesia*.

[30] Guerci P, Claudot JL, Novy E, Settembre N, Lalot JM, Losser MR (2018). Immediate postoperative plasma neutrophil gelatinase-associated lipocalin to predict acute kidney injury after major open abdominal aortic surgery: A prospective observational study. *Anaesthesia, Critical Care & Pain Medicine*.

[31] Pilarczyk K, Panholzer B, Huenges K, Salem M, Jacob T, Cremer J (2022). Prediction of acute kidney injury by cystatin c and [timp-2]*[igfbp7] after thoracic aortic surgery with moderate hypothermic circulatory arrest. *Journal of Clinical Medicine*.

[32] Wang J, Yang B, Liu M, You T, Shen H, Chen Y (2022). Serum cystatin C is a potential predictor of short-term mortality and acute kidney injury in acute aortic dissection patients: a retrospective cohort study. *Journal of Thoracic Disease*.

[33] Averdunk L, Rückbeil MV, Zarbock A, Martin L, Marx G, Jalaie H (2020). SLPI - a Biomarker of Acute Kidney Injury after Open and Endovascular Thoracoabdominal Aortic Aneurysm (TAAA) Repair. *Scientific Reports*.

[34] Wu Q, Li J, Chen L, Yan LL, Qiu Z, Shen Y (2020). Efficacy of interleukin-6 in combination with D-dimer in predicting early poor postoperative prognosis after acute stanford type a aortic dissection. *Journal of Cardiothoracic Surgery*.

[35] Zhang Y, Lan Y, Chen T, Chen Q, Guo Z, Jiang N (2022). Prediction of Acute Kidney Injury for Acute Type A Aortic Dissection Patients Who Underwent Sun’s Procedure by a Perioperative Nomogram. *Cardiorenal Medicine*.

[36] Kim WH, Lee SM, Choi JW, Kim EH, Lee JH, Jung JW (2013). Simplified clinical risk score to predict acute kidney injury after aortic surgery. *Journal of Cardiothoracic and Vascular Anesthesia*.

[37] Burne MJ, Daniels F, El Ghandour A, Mauiyyedi S, Colvin RB, O’Donnell MP (2001). Identification of the CD4(+) T cell as a major pathogenic factor in ischemic acute renal failure. *The Journal of Clinical Investigation*.

[38] Zhang MZ, Wang X, Wang Y, Niu A, Wang S, Zou C (2017). IL-4/IL-13-mediated polarization of renal macrophages/dendritic cells to an M2a phenotype is essential for recovery from acute kidney injury. *Kidney International*.

[39] Tam FW, Smith J, Karkar AM, Pusey CD, Rees AJ (1997). Interleukin-4 ameliorates experimental glomerulonephritis and up-regulates glomerular gene expression of IL-1 decoy receptor. *Kidney International*.

[40] Moledina DG, Mansour SG, Jia Y, Obeid W, Thiessen-Philbrook H, Koyner JL (2019). Association of T Cell-Derived Inflammatory Cytokines With Acute Kidney Injury and Mortality After Cardiac Surgery. *Kidney International Reports*.

[41] Liu KD, Altmann C, Smits G, Krawczeski CD, Edelstein CL, Devarajan P (2009). Serum interleukin-6 and interleukin-8 are early biomarkers of acute kidney injury and predict prolonged mechanical ventilation in children undergoing cardiac surgery: a case-control study. *Critical Care*.

[42] Greenberg JH, Zappitelli M, Jia Y, Thiessen-Philbrook HR, de Fontnouvelle CA, Wilson FP (2018). Biomarkers of AKI Progression after Pediatric Cardiac Surgery. *Journal of the American Society of Nephrology*.

