# Supplementary material for: A Novel Predictive Model for Acute Kidney Injury Following Surgery of the Aorta
Source: Rev Cardiovasc Med. 2024 Feb 4;25(2):54. doi: 10.31083/j.rcm2502054 (PMC11263166; doi:10.31083/j.rcm2502054)
Supplement: Supplementary file 1 [file 2153-8174-25-2-054-s1.docx]

**Supplementary Materials**

**Supplementary Table 1. The stages of AKI according to AKIN guideline.**

|  | The changes of Scr from baseline creatinine |
| --- | --- |
| Stage 1 | The highest Scr within 48 hours increased by over 0.3 mg/dl (≥ 26.5 μmol/l), or increased to 1.5-to-2 fold from baseline. |
| Stage 2 | The highest Scr within 48 hours increased to > 2-to-3 fold from baseline. |
| Stage 3 | The highest Scr within 48 hours increased to > 3 fold from baseline or Scr increased by more than 4.0 mg/dl (≥ 354μmol/l). |
|  |  |

AKIN, Acute Kindney Injury Network; Scr, Serum creatinine.

**Supplementary Table 2. The distribution of surgical procedures.**

| Operations | Number | Propotion（%） |
| --- | --- | --- |
| Bentall procedure | 27 | 17.30 |
| David procedure | 5 | 3.20 |
| Wheat procedure | 15 | 9.61 |
| Sun's procedure | 42 | 26.92 |
| Ascending aorta and total arch replacement | 10 | 6.41 |
| Hybrid total arch replacement | 18 | 11.53 |
| Ascending aorta and hemi-arch replacement | 3 | 1.92 |
| Open descending thoracic aorta replacement | 4 | 2.56 |
| Open thoraco-abdominal aorta replacement | 4 | 2.56 |
| Open repair of abdominal aortic aneurysms | 1 | 0.64 |
| Bentall procedure + Sun's procedure | 7 | 4.48 |
| Bentall procedure + Hybrid total arch replacement | 3 | 1.92 |
| Bentall procedure + Ascending aorta and hemi-arch replacement | 2 | 1.28 |
| Wheat procedure + Sun's procedure | 9 | 5.76 |
| Wheat procedure + Ascending aorta and hemi-arch replacement | 4 | 2.56 |
| David procedure + Sun's procedure | 1 | 0.64 |
| Wheat procedure + Ascending aorta and total arch replacement | 1 | 0.64 |
| Surgery simply referring to aortic root | 47 | 30.13 |
| Surgery simply referring to aortic rach | 18 | 11.54 |
| Surgery simply referring to descending aorta | 9 | 5.76 |
| Combined surgery | 82 | 52.57 |
| Total | 156 | 100 |

The Sun’s procedure refers to total arch replacement using a tetrafurcate vascular graft in combination with implantation of a special stented graft into the descending aorta. Combined surgery, procedures that involved at least two sites among the aortic root, ascending aorta, arch, and descending aorta.
